# Serological Diagnosis of Autoimmune Bullous Skin Diseases

**DOI:** 10.3389/fimmu.2019.01974

**Published:** 2019-08-20

**Authors:** Sandra Saschenbrecker, Ingolf Karl, Lars Komorowski, Christian Probst, Cornelia Dähnrich, Kai Fechner, Winfried Stöcker, Wolfgang Schlumberger

**Affiliations:** Institute for Experimental Immunology, Euroimmun AG, Lübeck, Germany

**Keywords:** autoantibody, autoimmune bullous dermatosis, biochip, ELISA, indirect immunofluorescence, pemphigoid, pemphigus, serology

## Abstract

Autoimmune bullous dermatoses (AIBD) encompass a variety of organ-specific autoimmune diseases that manifest with cutaneous and/or mucosal blisters and erosions. They are characterized by autoantibodies targeting structural proteins of the skin, which are responsible for the intercellular contact between epidermal keratinocytes and for adhesion of the basal keratinocytes to the dermis. The autoantibodies disrupt the adhesive functions, leading to splitting and blister formation. In pemphigus diseases, blisters form intraepidermally, whereas in all other disease types they occur subepidermally. Early identification of autoimmune bullous dermatoses is crucial for both treatment and prognosis, particularly as regards tumor-associated disease entities. The diagnosis is based on clinical symptoms, histopathology, direct immunofluorescence to detect antibody/complement deposits, and the determination of circulating autoantibodies. The identification of various target antigens has paved the way for the recent development of numerous specific autoantibody tests. In particular, optimized designer antigens and multiplex test formats for indirect immunofluorescence and ELISA have enhanced and refined the laboratory analysis, enabling highly efficient serodiagnosis and follow-up. This review elaborates on the current standards in the serological diagnostics for autoimmune bullous dermatoses.

## Introduction

**Figure 1:**
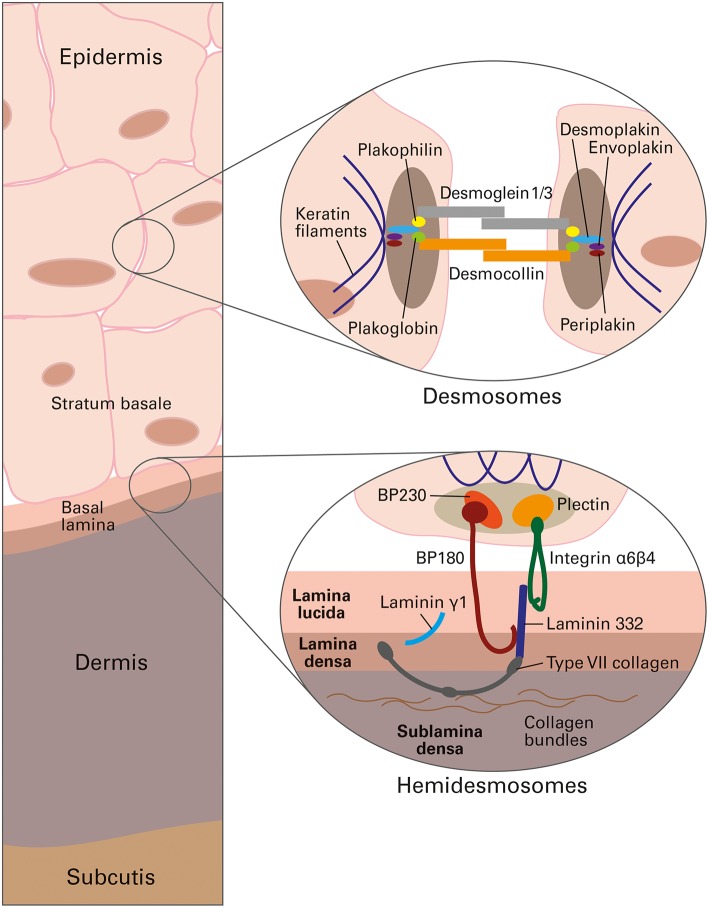
Schematic presentation of human skin, depicting the targets of autoantibodies in autoimmune bullous dermatoses, reproduced from Gosink and Schlumberger, MEDLAB Magazine 2016 (1) with permission of MEDLAB Magazine. Top circle: antigenic structural components of desmosomes, which interconnect the cytoskeletons of neighboring keratinocytes in the epidermis. Bottom circle: antigenic structural components of hemidesmosomes, which anchor the cells of the epidermal stratum basale in the underlying basal lamina at the dermal-epidermal junction.

Autoimmune bullous dermatoses (AIBD) are associated with autoantibodies that bind to structural proteins in the skin and mucous membranes, which are components of desmosomes (e.g., desmogleins, desmocollins, plakins) and hemidesmosomes (e.g., BP180, BP230, plectin, α6β4 integrin, laminin 332, laminin γ1, type VII collagen) [Figure 1, (1)]. These autoimmune reactions interfere with intercellular connections and anchoring mechanisms within the epidermis and dermal-epidermal junction, leading to the separation of skin layers and the formation of blisters and/or erosions (2, 3). The most important AIBD types and corresponding target antigens are summarized in Table 1.

**Table 1:** Autoantibody specificities in autoimmune bullous dermatoses.

**Blistering**	**Disease**	**Ig type**	**Target antigen^a^^,^^b^^,^^c^**
Intra-epidermal	Pemphigus foliaceus	IgG	**Dsg1**
	Pemphigus vulgaris	IgG	**Dsg3**, Dsg1
	IgA pemphigus	IgA	**Dsg1, Dsg3**, ***Dsc1**, Dsc2, Dsc3*
	Paraneoplastic pemphigus	IgG	**Dsg3**, Dsg1, **envoplakin**, ***periplakin**, desmoplakin I, desmoplakin II, epiplakin, plectin*, BP230, *Dsc1, Dsc2, Dsc3, α2-macroglobulin-like protein 1*
	Pemphigus vegetans	IgG	Dsg3, Dsg1, *Dsc3*
	Pemphigus erythematosus	IgG	Dsg1
	Pemphigus herpetiformis	IgG	**Dsg1**, Dsg3, *Dsc1, Dsc3*
	Drug-induced pemphigus	IgG	**Dsg1**, Dsg3
Sub-epidermal	Bullous pemphigoid	IgG	BP180*, BP230
	Pemphigoid gestationis	IgG	BP180*, BP230
	Linear IgA dermatosis	IgA	BP180^**/***^, BP180*, BP230
	Mucous membrane pemphigoid	IgG/IgA	BP180^****^, BP180^*^, ***laminin 332***, BP230, *α6β4 integrin*
	Anti-laminin γ1/p200 pemphigoid	IgG	***Laminin γ1 (p200)***
	Lichen planus pemphigoides	IgG	BP180^*^, BP230
	Epidermolysis bullosa acquisita	IgG	**Type VII collagen**
	Dermatitis herpetiformis	IgA (IgG)	**Epidermal transglutaminase**, tissue transglutaminase, endomysium, deamidated gliadin

a *Main target antigens are indicated in bold*.

b *Parameters for which commercial monospecific detection assays are not available are indicated in italics*.

c Immunodominant regions: Dsg1: N-terminal ectodomain, Dsg3: N-terminal ectodomain, BP180: ^*^NC16A/^**^LABD97/^***^LAD-1/^****^C-terminal epitopes; BP230: globular C-terminal domain; type VII collagen: N-terminal NC1 domain.

### Intraepidermal Blistering Diseases

In pemphigus diseases, the autoimmune system targets primarily the cadherin-type transmembrane adhesion molecules desmoglein (Dsg) 1 and 3. Desmogleins, together with desmocollins, provide cohesion between epidermal keratinocytes, and are linked intracellularly to the intermediate filament network via different types of plakins (Figure 1). In response to autoantibody binding, cell metabolism, intracellular signaling and desmosome structure are subject to alterations that cause the loss of cell-to-cell adhesion (acantholysis) and intra-epidermal split formation, resulting in flaccid blisters and erosions in the skin and/or mucous membranes (4, 5).

The clinical phenotype of pemphigus (i.e., the site of blister formation) is determined by the underlying antibody profile and the normal tissue distribution of Dsg1 and Dsg3. Dsg1 is predominantly expressed on the surface of the epidermis, whereas Dsg3 accumulates mainly in deeper epidermal layers and in the mucous membranes. As pemphigus foliaceus (PF) is associated only with IgG autoantibodies against Dsg1, blistering is confined to the upper skin, while there is no apparent mucosal involvement. In pemphigus vulgaris (PV), Dsg3 is the major autoantigen, but 50–60% of patients have additional autoantibodies to Dsg1. PV manifests as three different subtypes (2): [i] in mucosal-dominant PV, antibodies are restricted to Dsg3 and induce blisters in deep layers of the oral mucosa; [ii] patients with mucocutaneous PV exhibit reactivity against both Dsg1 and Dsg3 and show involvement of the epidermis in addition to the mucous membranes; [iii] the cutaneous PV type is less frequent and associated with blistering in deep epidermal layers owing to anti-Dsg1 and pathogenically weak anti-Dsg3. In contrast to PF, acantholysis takes place in the lower skin layers (2, 6–11). PV is the most frequent intraepidermal AIBD, accounting for 80% of all pemphigus cases and, for the most part, affecting middle-aged and elderly persons.

In patients with PV, numerous autoantibodies have been identified that target other structural and metabolic proteins, such as desmocollins (Dsc) 1 and 3, muscarinic and nicotinic acetylcholine receptors, mitochondrial antigens, thyroid peroxidase, hSPCA1, plakophilin 3, plakoglobin, and E-cadherin. Studies on the pathogenic role of some of these non-Dsg autoantibodies suggest that they synergistically complement the classic effects of anti-Dsg autoantibodies in the complex process of pemphigus pathogenesis (12–15).

IgA pemphigus (also referred to as “intercellular IgA dermatosis”) (16, 17) has been found in association with serum IgA reactivity against desmosomal cadherins, i.e., Dsc1, Dsc2, Dsc3, Dsg1, and Dsg3 (18–21).

Paraneoplastic pemphigus is a life-threatening form of pemphigus that is associated with a neoplasm (e.g., non-Hodgkin's lymphoma, chronic lymphocytic leukemia, Castleman tumor, thymoma, sarcoma, Waldenstrom's macroglobulinemia) (22). Pathogenesis is based on a combination of humoral and cellular autoimmune responses (23). Circulating autoantibodies are directed against multiple antigens, including predominantly plakins (envoplakin, periplakin, desmoplakin I, desmoplakin II, epiplakin, plectin, BP230), but also cadherins (Dsg3, Dsg1, Dsc1, Dsc2, Dsc3), α2-macroblobulin-like 1 (24–34). Due to their high specificity (91–100%), anti-envoplakin autoantibodies are considered an important diagnostic marker for paraneoplastic pemphigus (35–38).

In addition, the pemphigus group includes several (atypical) variants, such as pemphigus vegetans (39), pemphigus erythematosus (40, 41), pemphigus herpetiformis (42), endemic pemphigus (fogo selvage etc.) (43), and drug-induced pemphigus (44, 45).

### Subepidermal Blistering Diseases

The heterogenous group of pemphigoid diseases is characterized by subepidermal blister formation, which can occur in the skin and mucous membranes (3). Circulating autoantibodies target components of the dermal-epidermal junction (Figure 1) (46). As the targeted hemidesmosomal proteins and structural filaments provide contact between the epidermal cells and the basement membrane, the autoimmune reactions cause the epidermis to peel away from the underlying dermis.

Bullous pemphigoid (BP) is the most common AIBD and occurs primarily in the elderly (onset in the late 70s) (47). It manifests with tense, bulging blisters on inflamed or non-inflamed skin, while mucous membranes are rarely affected. Patient serum contains IgG targeting mainly the hemidesmosomal proteins BP180 and BP230. BP180 is a transmembrane glycoprotein whose major immunogenic epitopes are located in the extracellular 16th non-collagenous domain (BP180-NC16A) (48). Due to their high prevalence, anti-BP180 autoantibodies represent the most important serological marker for BP. BP230 is a cytoplasmic protein which interacts with BP180. Its globular C-terminal domain mediates the attachment of keratin filaments to the hemidesmosomal plaque and contains the majority of immunoreactive sequences (49). Anti-BP230 positivity occurs in a subset of anti-BP180 negative BP patients, making it an important additional marker (50–54). Many BP sera also exhibit reactivity against antigenic sites outside the immunodominant domains of BP180 and BP230, which should be addressed in those BP patients unreactive with the immunodominant domains (<10%) (52, 55).

Pemphigoid gestationis is a manifestation of BP occurring in pregnant women and in puerperium, presenting with urticarial plaques and/or tense blisters. BP180-NC16A is the main target (90%) of autoantibodies in patients with pemphigoid gestationis, while anti-BP230 reactivity is less prevalent (56, 57).

The serological hallmark in linear IgA dermatosis is anti-basement membrane reactivity of class IgA autoantibodies recognizing the 120 kDa ectodomain fragment of BP180, referred to as linear IgA disease antigen 1 (LAD-1) and a derivative thereof (linear IgA bullous disease antigen of 97 kDa, LABD97) (58–61). A small proportion of sera recognizes BP180-NC16A or BP230 (62–64).

Mucous membrane pemphigoid affects one or more mucous membranes (e.g., oral, ocular, genital, anal) and may also involve the skin. Patients exhibit low-titer IgG/IgA autoantibodies directed against components of the basement membrane zone, with BP180 and laminin 332 presenting the two major targets (65). Anti-BP180 reactivity is not only directed against the NC16A domain, but also against C-terminal extracellular epitopes (66–69). The identification of anti-laminin 332 positive patients is vitally important as they have an increased relative risk for cancer, with malignancies occurring in about 25–30% of cases (70–73). Furthermore, patients with mucous membrane pemphigoid may exhibit increased IgG/IgA against BP230 (74, 75) or α6β4 integrin, the latter indicating the presence of ocular lesions (76, 77).

In anti-laminin γ1/p200 pemphigoid, tense blisters can be found on erythematosus or normal skin, with a high tendency to affect acral surfaces (78, 79). The associated autoantibodies target a 200-kDa basement membrane protein, referred to as laminin γ1 (80, 81).

Lichen planus pemphigoides emerges with bullous skin lesions in conjunction with lichen planus. Compared to BP, this disease has a much lower incidence, affects younger patients (onset 40–50 years), is usually less severe and arises mainly on the limbs (82). Serum reactivity is preferentially directed against C-terminal epitopes in the immunodominant NC16A domain of BP180 (83, 84).

Epidermolysis bullosa acquisita (EBA) is a rare, subepidermal blistering disease that can occur at any age. Patients suffer from chronic inflammation, blistering and scarring of the skin and mucous membranes. (85, 86). A characteristic feature is the presence of autoantibodies directed against type VII collagen, the main constituent of anchoring fibrils at the dermal-epidermal junction, with the major antigenic epitopes located within the amino-terminal non-collagenous domain (NC1) (87–90).

Dermatitis herpetiformis (Duhring's disease) is the cutaneous manifestation of coeliac disease (sprue, gluten-sensitive enteropathy), affecting about 10% of coeliac patients. It is characterized by blisters forming in deeper (subepidermal) layers of the skin, while the mucous membranes do not show any blistering. The targets of circulating IgA antibodies are epidermal/tissue transglutaminase, endomysium, and deamidated gliadin (91–95). Since the underlying gluten-sensitive enteropathy is frequently associated with selective IgA deficiency, the additional determination of class IgG antibodies can be diagnostically indicated (96).

## Diagnostic Approach

The diagnosis of AIBD is detailed in recent publications (97–104). Commonly recommended approaches are based on several pillars that cover symptomatic evaluation and laboratory tests.

Firstly, the clinical characteristics have to be determined, including patient history, physical examination, and assessment of the disease activity (105).

Secondly, histopathology is performed on lesional skin or mucosal biopsy. Although of limited diagnostic value, the observation of intra-/subepidermal cleavage and inflammatory infiltrates can give a first information for differentiation between pemphigus and pemphigoid diseases.

Thirdly, direct immunofluorescence (DIF) microscopy using cryosections of perilesional biopsy specimens is performed to detect tissue-bound autoantibodies. This method is still the diagnostic gold standard, with a sensitivity in the range of 82–91% and a specificity of 98% (106–110), but it provides only limited information on the target antigens. DIF microscopy narrows down the diagnosis according to the deposited Ig subclass and binding pattern (103). For example, intercellular deposition of IgG and/or C3 in the epidermis is characteristic of PV, PF, and paraneoplastic pemphigus. By contrast, linear binding of IgG and/or C3 at the dermal-epidermal junction can be found in pemphigoid diseases, with further differentiation options based on the serration pattern (u-serration vs. n-serration) (103, 111). Granular IgA deposits along the basement membrane zone and at the dermal papillae tips are observed in dermatitis herpetiformis.

The fourth pillar addresses the serological detection and differentiation of circulating autoantibodies. Serology has the advantage of being minimally invasive, which is particularly helpful in cases where biopsy specimens cannot be obtained (children, uncooperative adults). In many cases, serological testing may even suffice to establish the diagnosis in conjunction with a compatible clinical picture (112). Serum analysis relies on indirect immunofluorescence (IIF) microscopy using native tissue sections and recombinant proteins as substrates. Recombinant antigens are also applied in immunoblot or immunoprecipitation analyses and in enzyme-linked immunosorbent assays (ELISA), the latter having additional relevance to the monitoring of disease activity. Conventionally, the serological diagnosis of AIBD follows a multi-step approach that is based on initial IIF screening using one or two tissue substrates, followed by individual antigen-specific assays (ELISA, immunoblot) that correspond to the clinical suspicion and the IIF screening results. Meanwhile, alternative approaches for highly efficient and expeditious testing are available utilizing multiparametric analysis tools (113, 114). In clinical practice, routine serological results should be interpreted with care, taking into consideration the possibility of discrepancies between IIF and ELISA or negative serology in biopsy-proven patients. Assay results may even be positive in cases without other laboratory or clinical evidence of pemphigus (109, 115). Such inconsistent findings complicate decision-making, bearing the risk of misdiagnosis. Where available, alternative serological methods (e.g., keratinocyte binding assay) may provide additional information to ascertain or rule out a diagnosis, especially when no biopsy is available (115).

Early diagnosis and differentiation of AIBD is crucial for the initiation of an appropriate treatment. In most AIBD entities (e.g., BP, linear IgA disease, anti-laminin γ1/p200 pemphigoid), systemic corticosteroids in combination with further immunosuppressants/-modulants are sufficient to induce clinical remission, whereas treatment of pemphigus remains challenging as reflected by a mortality of 8–42% in mucocutaneous PV (116). However, prognosis has improved due to the development of new therapy options, including immunoadsorption, intravenous immunoglobulins, and anti-CD20 monoclonal antibodies (2, 112, 116–124). In paraneoplastic pemphigus and anti-laminin 332 mucous membrane pemphigoid, the disease prognosis may be unfavorable due to associated neoplasia in 100% and in up to 30% of cases, respectively (73, 125).

## Serological Screening Using Tissue Substrates in IIF

IIF microscopy using tissue substrates has traditionally been performed as a standard method for the detection of autoantibodies in AIBD. Due to their high sensitivity, these substrates have priority for screening purposes. However, they do not allow definite determination of the autoantibodies' specificity (e.g., differentiation between anti-Dsg1 and anti-Dsg3).

### Esophagus

**Figure 2:**
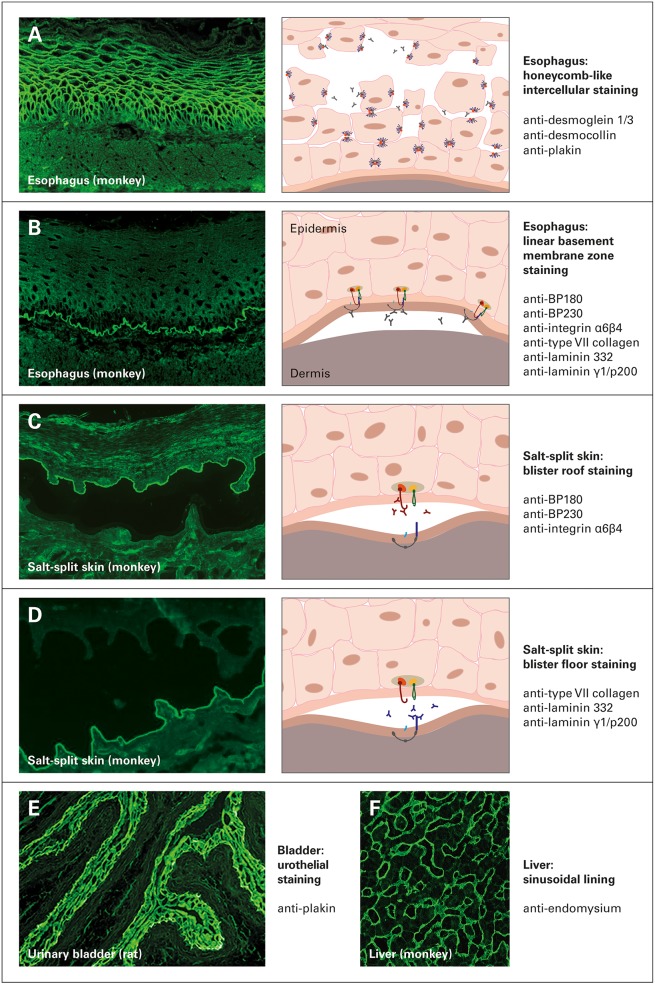
Indirect immunofluorescence staining of different tissue substrates in autoimmune bullous dermatoses. **(A,B)** Monkey esophagus, **(C,D)** monkey salt-split skin, **(E)** rat urinary bladder and **(F)** monkey liver. Graphics and annotations on the right indicate the staining patterns and possible underlying reactivities, reproduced and modified from Euroimmun customer leaflet, with permission of Euroimmun, Germany.

Esophagus from monkey or guinea pig is a highly sensitive substrate. Two characteristic immunofluorescence patterns can be differentiated on this tissue. [i] Pemphigus-specific autoantibodies result in a honeycomb-like fluorescence of the intercellular substance in the stratum spinosum. These autoantibodies are directed against prickle cell desmosomes, reacting with surface antigens of keratinocytes (Figure 2A). [ii] A fine linear staining between the stratum basale and the connective tissue is caused by anti-basement membrane zone autoantibodies, which are associated with pemphigoid diseases or EBA (Figure 2B).

When pemphigus serum is applied, monkey esophagus yields a sensitivity of 81–100% and a specificity of 89–100%, making it the optimal substrate in the screening for intercellular antibodies in suspected cases of PV and PF (113, 126–132). This substrate has often been reported to be more sensitive for PV than for PF as monkey esophagus is a mucosal tissue with high expression of Dsg3, the major target in PV, in contrast to lower Dsg1 expression (132). The predictive value of a negative test result is highly reliable to exclude the diagnosis of pemphigus, and false-positive results (intercellular staining of non-pemphigus sera) is not associated with an increased risk of developing pemphigus subsequently (132). In BP, 68–73% of cases were reported positive for anti-basement zone antibodies, at a specificity of 97% (51, 106, 133). It should be taken into account that antibodies against blood group antigens A and B may lead to unspecific desmosome fluorescence on monkey esophagus, potentially leading to false positive results. As this interference may account for up to 10% of healthy blood donors, adsorption reagents should be applied in suspected cases (e.g., blocking with soluble A/B antigens or with red blood cells from an AB-positive donor) (134).

### Salt-Split Skin

Skin, in which partial dermal-epidermal splitting is induced by incubation with a 1 M NaCl solution, presents the IIF substrate of choice when screening for autoantibodies in subepidermal AIBD (135, 136). It is optimally suited for the detection of anti-basement membrane zone autoantibodies, as reflected by a reported sensitivity of 73–96% and a specificity of 97% (106, 108, 133, 137). In addition, it allows the differentiation between autoantibodies with different antigenic binding properties. Anti-BP180, anti-BP230, and anti-α6β4 integrin stain the epidermal side of the artificial split (blister roof), as detectable in BP, pemphigoid gestationis, linear IgA dermatosis, and anti-BP180-type mucous membrane pemphigoid (Figure 2C). In contrast, anti-type VII collagen, anti-laminin 332, and anti-laminin γ1 bind along the dermal side of the split (blister floor), pointing toward EBA, anti-laminin-332-type mucous membrane pemphigoid, and anti-laminin γ1/p200 pemphigoid, respectively (Figure 2D) (100, 138).

### Urinary Bladder

In suspected cases of paraneoplastic pemphigus, IIF on rat (or monkey) urinary bladder is performed to detect autoantibodies against plakins and to distinguish paraneoplastic pemphigus from other pemphigus diseases. As envoplakin, periplakin, and desmoplakins (but not Dsg1 and Dsg3) are highly expressed in bladder tissue, and owing to the high specificity of this substrate (74% sensitivity, 99–100% specificity), positive IgG reactivity with the urothelium is considered a diagnostic indication of paraneoplastic pemphigus (38, 139) (Figure 2E). However, negative IIF on bladder does not exclude the diagnosis of paraneoplastic pemphigus and should entail other serological techniques (36, 38).

### Liver

Tissue sections of primate liver are best suited to visualize autoantibodies (IgA) against endomysium in dermatitis herpetiformis. Positive reactivity is indicated by a fluorescent filamentous lining of the intralobular sinusoids (140) (Figure 2F).

## Antigen-Specific Serological Assays

The identification of the autoantibodies' target antigens can be accomplished using monospecific IIF, ELISA and/or immunoblot tests. For this purpose, many antigenic substrates have been made available by means of recombinant expression systems. By selecting only immunoreactive epitopes and deleting domains that cause unspecific reactions, the sensitivity and specificity of the resulting assay can often be improved (141). For example, a recombinant tetramer of the immunodominant NC16A domain of BP180, termed BP180-NC16A-4X, was designed to multiply the number of antibody binding sites per molecule, thereby optimizing the immunoreactivity and diagnostic efficiency in BP serology. A second example concerns a gliadin-analogous fusion peptide (GAF-3X), which contains three repetitive modified copies of formerly described peptides recognized by autoantibodies in most patients with coeliac disease and dermatitis herpetiformis (142). After expression and purification from *Escherichia coli*, those two antigens were applied in IIF and ELISA (142–145).

### Recombinant Monospecific Substrates in IIF

**Figure 3:**
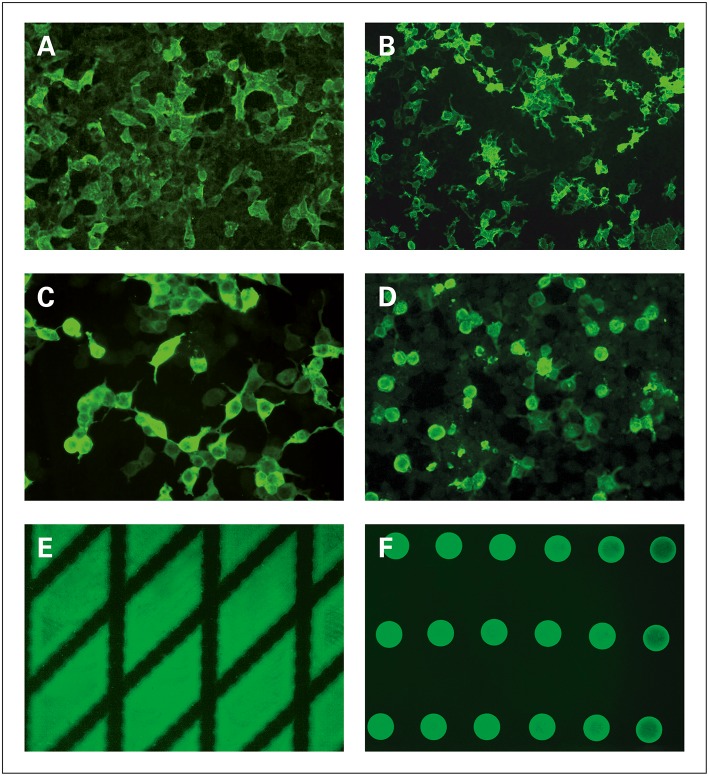
Detection and differentiation of autoantibodies in autoimmune bullous dermatoses using monospecific substrates for BIOCHIP-based indirect immunofluorescence, reproduced (in part) from Gosink and Schlumberger, MEDLAB Magazine 2016 (1) and from Gosink, MEDLAB Magazine 2013 (148) with permission of MEDLAB Magazine. **(A–D)** Substrates based on human embryonic kidney (HEK293) cells expressing recombinant immunodominant antigen domains: **(A)** Dsg1 (ectodomain), **(B)** Dsg3 (ectodomain), **(C)** BP230gC (globular C-terminal domain), **(D)** type VII collagen (NC1 domain). **(E,F)** Substrates generated by spotting purified recombinant protein: **(E)** BP180-NC16A-4X (tetrameric NC16A domain), **(F)** GAF-3X (trimeric deamidated gliadin-analogous fusion peptide).

Compared to classic tissue sections that contain a multitude of different antigens and sometimes require specialist knowledge for reliable interpretation, recombinant substrates considerably simplify IIF evaluation and may allow a *prima vista* differentiation between AIBD-associated diseases. Recombinant IIF assays are based on BIOCHIP technology (Euroimmun, Lübeck, Germany), in which the substrates are coated onto millimeter-sized BIOCHIPs and arranged on the reaction fields of microscope slides. The slides are incubated using the Titerplane technique, which provides parallel incubation of multiple samples under standardized, identical conditions (146). Two types of recombinant IIF substrates can be distinguished:

In the first case, the target antigen is expressed in the human cell line HEK293, which provides authentic conformational folding and post-translational modification (141, 147). Since transfected and mock-transfected control cells are coated onto the BIOCHIPs side by side, it is straightforward to distinguish true-positive sera containing antigen-specific antibodies (smooth to fine granular cytosolic fluorescence only in the subset of transfected cells) from sera reacting against other cell components (nuclear or cytoplasmic staining of all cells). Available recombinant cell-based substrates for AIBD serology include Dsg1, Dsg3, BP230, and type VII collagen (Figures 3A–D) (144, 149).

In the second case, purified recombinant antigens (e.g., BP180-NC16A-4X and GAF-3X) are coated directly onto the BIOCHIPs. If a positive serum sample is applied, the antigenic areas will fluoresce in a particular pattern (e.g., diamonds or circles) against a dark background (Figures 3E,F).

### Multiparametric BIOCHIP Mosaics in IIF

**Figure 4:**
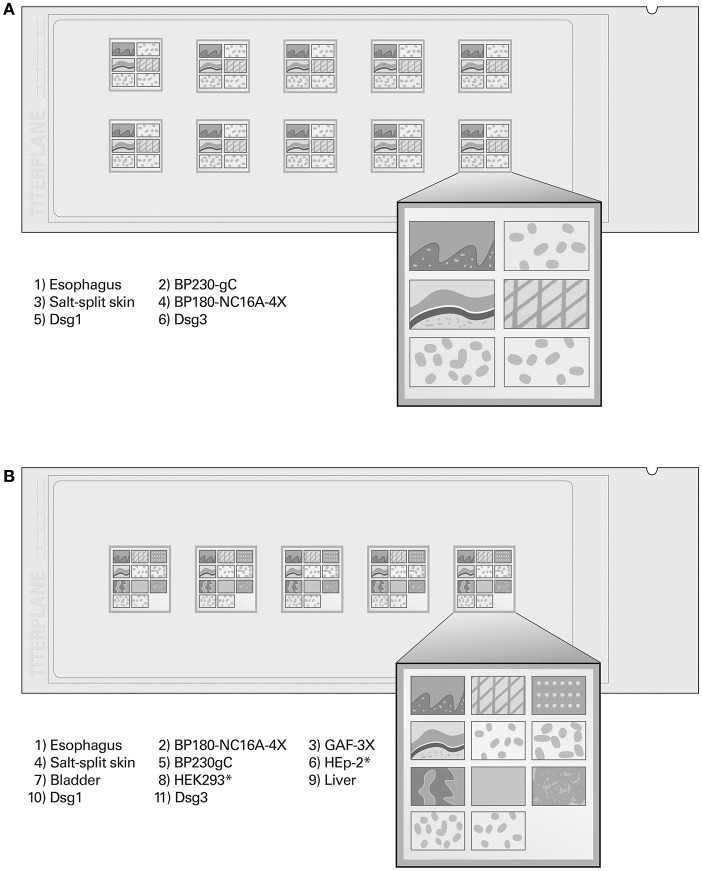
BIOCHIP mosaics for simultaneous screening and monospecific confirmation of autoantibodies using indirect immunofluorescence, modified from Gosink, MEDLAB Magazine 2013 (148) with permission of MEDLAB Magazine. **(A)** “Dermatology Mosaic 7” (six substrates per reaction field). **(B)** “Dermatology Mosaic 11” (11 substrates per reaction field for extended analysis including paraneoplastic pemphigus and dermatitis herpetiformis). As indicated, the BIOCHIPs are coated with tissue sections (monkey esophagus, salt-split skin, liver, rat urinary bladder), HEK293 cells expressing recombinant antigens (Dsg1, Dsg3, BP230gC), or spots of purified recombinant antigen (BP180-NC16A-4X, GAF-3X). ^*^HEp-2 and mock-transfected HEK293 cells serve as negative control substrates.

The recombinant monospecific IIF substrates can be analyzed side by side with classic tissue sections in standardized BIOCHIP mosaics (Euroimmun; Figure 4). The combination of different substrates in the same test field allows autoantibody screening and confirmatory discrimination to be carried out in a single incubation, thus facilitating differential diagnosis among the various types of AIBD. Particularly in diagnostically difficult cases, this multiparametric technique is cost- and time-effective compared to the conventional multi-step approach (113, 150).

Several studies have been performed on the diagnostic performance of the mosaic-based IIF technique. Cumulative findings indicate that this method is highly sensitive and specific for pemphigus and BP (150–152). For example, monkey esophagus yielded sensitivities of 83–100% (PV), 98% (PF), and 69% (mixed pemphigus panel), with specificities in the range of 89–100%. Anti-Dsg1 was detectable with a sensitivity of 19–52% (PV), 90% (PF), and 38% (mixed pemphigus panel) and a specificity of ≥99%. The sensitivity of anti-Dsg3 detection amounted to 98–100% (PV) and 87% (mixed pemphigus panel), with specificities ranging from 97 to 100% (113, 152–154). In BP, basement membrane zone staining on esophagus and/or salt-split skin provided a sensitivity and specificity of 50–99 and 77–100%, respectively. The sensitivity and specificity for anti-BP180 detection were reported to be 83–100 and 97–100%, respectively, and for anti-BP230 detection 30–67 and 97–100%, respectively (113, 144, 152, 154, 155). van Beek et al. (113) compared the performance of the “Dermatology Mosaic 7” (Figure 4A) with the conventional multi-step procedure (156). Between both approaches, high diagnostic agreement (94%, kappa 0.88–0.97) was observed. In <5% of the cases, final diagnosis could only be made by using the appropriate assays within the multi-step approach, because additional substrates would have to be added to the standard IIF mosaic for these rare disorders. Meanwhile, however, further mosaics adjusted to the diagnosis of particular AIBD types have been evaluated, including the detection of [i] anti-BP180 in pemphigus gestationis (100% sensitivity, 100% specificity) (157), [ii] anti-type VII collagen NC1 in EBA (92% sensitivity, 100% specificity) (149), [iii] anti-laminin 332 in mucous membrane pemphigoid (77–84% sensitivity, 100% specificity) (72), and [iv] anti-Dsc in atypical pemphigus variants (158). Tampoia et al. compared the concordance between results obtained by mosaic-based IIF vs. ELISA (MBL/Euroimmun) and found excellent agreements for the determination of anti-Dsg3 (kappa 0.97–1.00) and anti-BP180 (kappa 0.94–0.90) (154). Özkesici et al. reported correlations between mosaic-based IIF and ELISA (Euroimmun) of 85% (anti-Dsg1), 94% (anti-Dsg3), and 98% (anti-BP180) (152).

### Enzyme-Linked Immunosorbent Assays

ELISA systems based on recombinant target antigens are widely available and increasingly applied in the serological diagnosis of AIBD. They are used to confirm and differentiate autoantibody specificities, supporting the diagnostic attribution of AIBD subtypes. Moreover, ELISA allow quantitative measurement of antibody levels, enabling disease and therapy monitoring, as described below. In most cases, ELISA are sufficient to support the diagnosis and cheaper than other complex techniques. Further advantages include standardization, objective data, easy handling, automated processing, high throughput, and (for most parameters) commercial availability.

Commercial ELISA systems (MBL, Euroimmun) are available for the detection of autoantibodies against Dsg1 and Dsg3 in pemphigus (147, 159) and against envoplakin in paraneoplastic pemphigus (36). In pemphigoid diseases, commercial ELISA (MBL, Euroimmun) include BP180 (143, 160), BP230 (50, 54), and type VII collagen (149, 161). Importantly, the highest detection rate among BP patients is achieved by combining the ELISA results for anti-BP180 and anti-BP230 (87–100%), reflecting a diagnostic added value compared to mere anti-BP180 testing (50–54). Therefore, in cases with clinically suspected cases of BP, where anti-BP180 testing is negative, it is recommended to analyze serum reactivity against BP230 (97). Moreover, ELISA for the detection of autoantibodies against deamidated gliadin and transglutaminase (92) are available, supporting the diagnosis of dermatitis herpetiformis. Besides, less standardized in-house ELISA systems are applied in specialized laboratories, including rare parameters, such as anti-laminin γ1 (162), anti-desmocollin (20, 33), anti-laminin 332 (71, 163), and anti-BP180 (various forms) (52, 164). The diagnostic performance of commercial and in-house ELISA systems has been examined in numerous studies (Table 2) and discussed in reviews by Tampoia et al. (190) and Horvath et al. (165).

**Table 2:** Performance characteristics of reported ELISA systems for the detection of autoantibodies in autoimmune bullous dermatoses.

**ELISA^a^**	**Disease**	**Sensitivity^b^**	**Specificity^b^**	**References**
Anti-Dsg1^c^	Pemphigus foliaceus	96 – 100%	96–100%	(147, 159, 165–169)
Anti-Dsg3^c^	Pemphigus vulgaris	85–100%	96–100%	(147, 159, 165–173)
Anti-envoplakin^d^	Paraneoplastic pemphigus	63–83%	91–98%	(36–38)
*Anti-periplakin*	Paraneoplastic pemphigus	74%	96%	(36)
*Anti-desmocollin*	Paraneoplastic pemphigus	60%	NA	(33)
Anti-BP180^e^	Bullous pemphigoid	54–95%	90–100%	(50, 51, 53, 106, 143, 144, 160, 164, 165, 169, 173–181)
Anti-BP230^f^	Bullous pemphigoid	48–82%	65–99%	(50, 51, 53, 54, 106, 144, 165, 175–177, 182)
*Anti-laminin 332*	Mucous membrane pemphigoid	20–75%	84–96%	(71, 163)
*Anti-laminin γ1*	Anti-lamininγ1/p200 pemphigoid	69%	99%	(162)
Anti-type VII collagen^g^	Epidermolysis bullosa acquisita	86–100%	98–100%	(114, 149, 161, 183–186)
Anti-deamidated gliadin	Dermatitis herpetiformis, coeliac disease	84–95% (IgA)	86–93% (IgA)	(92, 145)
		80–99% (IgG)	93–94% (IgG)	
Anti-tissue transglutaminase	Dermatitis herpetiformis, coeliac disease	78–98%	96–99%	(92, 94, 145, 187–189)

a *Parameters for which commercial ELISA systems are not available are indicated in italics*.

b *Including performance data reported for commercial and in-house assays*.

c *Commercial assays employ the ectodomains of Dsg1 and Dsg3 after recombinant expression in baculovirus (MBL) or HEK293 cells (Euroimmun)*.

d *Commercial assay based on the N-terminal envoplakin 1–481 fragment (Euroimmun)*.

e *Commercial assays employ a single recombinant NC16A domain (MBL) or a tetramer of four NC16A domains to increase epitope exposure (BP180-NC16A-4X, Euroimmun)*.

f *Commercial assays employ recombinant protein of both N- and C-terminal parts of BP230 (MBL) or only a fragment of the C-terminal domain (BP230_2326−2649_, Euroimmun)*.

g *Commercial assays employ the NC1 and NC2 domains of type VII collagen (MBL) or only NC1 (Euroimmun)*.

Although being highly sensitive and specific, ELISA may produce positive results without clinical or other laboratory evidence.

### Multiparametric ELISA

In order to further improve and accelerate the routine serological diagnosis of AIBD, two profile ELISA systems have been developed that enable multiparametric antigen-specific testing for autoantibodies in adjacent wells of a microplate. By simultaneous processing of the diagnostically most relevant antigens, multiplex ELISA offer an alternative to IIF as serological first-line approach and to a multi-step single testing strategy.

Horvath et al. analyzed the diagnostic performance of the MESACUP Anti-Skin Profile (MBL), which covers five target antigens: Dsg1, Dsg3, BP180, BP230, and type VII collagen. They reported a 88% concordance with data obtained from the respective individual ELISA systems (MBL), resulting in sensitivities of 92% (anti-Dsg1, PF), 93% (anti-Dsg3, PV), 66% (anti-BP180, BP), 62% (anti-BP230, BP), and 81% (anti-type VII collagen, EBA), and specificities of 98–100% (165).

Van Beek et al. validated the Dermatology Profile ELISA (Euroimmun), comprising the same five parameters plus additional envoplakin. They demonstrated sensitivities of 95% (anti-Dsg1, PF), 100% (anti-Dsg3, PV), 95% (anti-BP180, BP), 60% (anti-BP230, BP), 93% (anti-type VII collagen, EBA), and 86% (anti-envoplakin, paraneoplastic pemphigus), and specificities in the range of 97–100% (114). These performance characteristics were also similar to those obtained with the individual ELISA (Euroimmun) (36, 54, 143, 147, 149). Comparison of the Dermatology Profile to the conventional multi-step approach yielded concordant results in 87%. Incongruent results were attributed to the lack of IgA detection and reactivity against antigens not included in the profile ELISA (114).

### Immunoblotting

Immunoblotting and immunoprecipitation help to determine rather rare autoantibodies (e.g., anti-laminin γ1, anti-laminin 332, anti-LAD-1, anti-α6β4 integrin, anti-desmoplakin, anti-type VII collagen) and are based on recombinant proteins or cell extracts (e.g., epidermis, dermis, cultured keratinocytes) (28, 69, 80, 191–193). These tests, however, are time-consuming and available only as in-house assays in specialized laboratories. They allow for highly specific autoantibody detection, but have proven inadequate for targets with mainly conformational epitopes, such as Dsg1 and Dsg3 (127, 141, 194–196). Immunoblotting for anti-Dsg is thus not recommended in the diagnosis of PF/PV (97). In contrast, there are patients suspected of having pemphigoid disease who show positive DIF results in the absence of autoantibody reactivity by commercial ELISA systems. In such cases, immunoblotting using antigenic fragments outside the immunodominant domains may provide diagnostically relevant information on the autoantibodies' target.

## Immunological Monitoring

Autoantibodies in several AIBD entities are directly pathogenic (4, 197–205). Their titers correlate with the disease activity over time, as reported for anti-Dsg1, anti-Dsg3 (147, 159, 166, 170, 206–209), anti-BP180 (51, 52, 143, 160, 210–214), and anti-type VII collagen (161, 183, 184, 215). By contrast, anti-BP230 reactivity appears not to fluctuate with changes in the clinical course of BP patients or only in a small subset of cases (50–52).

IIF evaluation is subjective and produces only semiquantitative data, based on serial serum dilutions, with titers depending on the type of substrate due to variable antigen expression levels. As opposed to this, ELISA provide objective and quantitative scores, which tend to reflect the disease activity better than IIF titers (130, 160). Therefore, ELISA testing is routinely used in many laboratories for the monitoring of disease activity. However, the relationship is not always perfect as there are cases of active disease with negative ELISA results and vice versa, as well as cases where antibody levels do not fluctuate in correlation with clinical activity (116, 209).

As an adjunct to the paramount clinical assessment, the follow-up of autoantibody titers has relevance for disease monitoring and can be helpful in therapeutic decisions, such as adjusting the dose of immunosuppressants. For example, when lesions have healed, decreasing or undetectable autoantibody levels may indicate dosage reduction or omission, respectively. In addition, relapses may be anticipated by the detection of increased autoantibody levels (99). However, the clinical judgement and the above-mentioned imperfections of the assays should always be taken into consideration (97, 116).

## Predictive Biomarkers for Disease Progression

Several molecules involved in, e.g., autoimmune and inflammatory responses in AIBD have recently been identified as potential biomarkers for disease development and outcome. The measurement of these biomarkers could help to adapt the duration and intensity of treatment in order to prevent the occurrence of relapses. The relevance of stratifying patients at risk of relapse, preferably at initiation of treatment, is reflected by a rate of about 30% of clinical relapses within the first year of treatment in patients with BP (216).

In BP patients, the value of monitoring anti-BP180 autoantibodies as a potential risk factor for relapse has been demonstrated. It was found that anti-BP180 IgG levels are significantly higher at baseline in patients who experience a relapse compared to non-relapse cases, whereas no such association was observed for anti-BP230 IgG, serum IgE, and peripheral eosinophils (217, 218). A multicenter prospective study demonstrated that the decrease in anti-BP180 titers during the first 60 days of treatment is lower in patients with relapse than in patients with ongoing remission. In addition, high anti-BP180 levels at day 150 provide high sensitivity for a relapse between days 150–360 of treatment (219). Cai et al. confirmed that increasing anti-BP180 IgG titers are associated with a decreasing remission rate (220). Along with anti-BP180, the detection of autoantibodies against type VII collagen may help to stratify BP patients based on the observation that about 40% of relapsing cases display positive and increasing anti-type VII collagen serum levels at the time of relapse (221).

In addition, the follow-up of molecules involved in inflammatory mechanisms can contribute to the prediction of BP outcome. Amongst others, this pertains to serum concentrations of the cytokines interleukin 17 (IL-17) and IL-23. The former shows significant decreases in patients with ongoing remission as well as constantly elevated levels prior to relapse, whereas the latter increases in early treatment stages in patients who later relapse (222). Similarly, an increased release of the chemokine CXCL10 favors BP relapse within the first year of treatment (223). These three inflammatory biomarkers all upregulate the secretion by leukocytes of matrix-metalloproteinase-9 (MMP-9), which was shown to decrease over time upon remission and to remain elevated in patients who relapse. Consequently, the follow-up of protease MMP-9 expression is regarded as another promising tool for the prediction of relapse in BP (222, 223). Also the serum concentrations of eosinophil cationic protein (ECP), as a measure of eosinophil activation, may help to predict a relapse as indicated by the absence of ECP alterations under treatment (224).

Moreover, an increased expression of the glucocorticoid receptor-beta in skin epithelial cells was suggested to be predictive of reduced treatment efficacy and increased risk of BP relapse (225). The presence of extensive disease (more than ten new blisters daily) at baseline and of neurological conditions associated with BP (e.g., dementia) may also play a role in the prediction of BP outcome (219).

In patients with PV, anti-Dsg3 positivity and, to a lesser extent, positive DIF results are predictors of relapse (226). Positivity for anti-Dsg1 and anti-Dsg3 was shown to provide high predictive values for the occurrence of relapses following treatment (209, 227). In addition, B-cell repopulation and low CD4+ T-cell count are associated with relapses in patients with pemphigus (227).

Altogether, this relatively new field warrants further investigation and holds the potential to benefit both clinicians and patients. In particular, to link cytokine/chemokine variations to clinical practice, large prospective studies will have to confirm the findings to date.

## Perspectives

Accurate diagnosis and discrimination of the different AIBD forms is crucial for therapeutic decisions and prognosis. Owing to highly sensitive and specific assays, it is estimated that a serological diagnosis can be made in about 90% of patients, subject to clinical expression (99, 112). Both the increasingly aging population and the constantly improved diagnostics cause a steady growth in the incidence of AIBD. In Germany, the annual incidence doubled within a decade, meanwhile amounting to about 25–30 cases per million inhabitants (112, 228). Hence, the continuous development and application of serological assays for known and yet unknown parameters will play a crucial role in the future. Patient management will further benefit from ongoing basic research on pathophysiological mechanisms and from clinical trials on forthcoming treatment options (229). For example, there is a growing number of data regarding the potential pathogenic role of IgE class autoantibodies in BP and the option of anti-IgE treatment (230–233).

## Author Contributions

SS performed the literature research, acquired and modified the figures, and wrote the first manuscript draft. IK, LK, CP, CD, KF, WSt, and WSc critically revised the manuscript. All authors have made a substantial, direct and intellectual contribution to the work, and approved it for publication.

### Conflict of Interest Statement

SS, IK, LK, CP, CD, and KF are employees of Euroimmun AG, a company that develops and manufactures immunoassays for the detection of disease-associated antibodies. WSt and WSc are board members of Euroimmun AG.
